# Employing bibliometrics and natural language processing (NLP) to analyse real-world applications of adverse drug reaction

**DOI:** 10.1016/j.rcsop.2025.100592

**Published:** 2025-03-17

**Authors:** Viola Savy Dsouza, Lada Leyens, Angela Brand

**Affiliations:** a Faculty of Health, Medicine, and Life Sciences (FHML), Maastricht University, Maastricht, the Netherlands; b Centre for Regulatory Science, Department of Health Information, Prasanna School of Public Health, Manipal Academy of Higher Education (MAHE), Manipal, Karnataka, India; c United Nations University - Maastricht Economic and Social Research Institute on Innovation and Technology, the Netherlands

**Keywords:** Adverse drug reaction, Pharmacovigilance, Natural language processing, Bibliometrics, Content analysis, Web application

## Abstract

**Introduction:**

Adverse Drug Reactions (ADRs) pose significant health and economic burdens, yet underreporting and inconsistent standards persist. Digital health innovations, particularly mobile and web-based ADR reporting applications, offer potential to enhance pharmacovigilance by improving data accuracy and patient-reported outcomes.

**Objectives:**

The study provides a comprehensive mapping of ADR mobile and web application literature, analysing publication trends, key contributors, and core themes through bibliometric and NLP-based content analysis.

**Methods:**

A systematic two-stage approach was applied to 289 Web of Science articles on ADR reporting applications. Bibliometric analysis explored publication trends, co-authorship networks, and keyword occurrences, while NLP-based topic modelling identified prevalent themes, ensuring thematic coherence and interpretability.

**Results:**

Bibliometric analysis showed a rise in ADR application-related publications, primarily from the United Kingdom, United States of America, and Switzerland. Content analysis identified ten key themes, including pharmacovigilance, chemotherapy adherence, and psychiatry research. A distinct focus on digital tools in ADR reporting and management was evident, with keywords such as “mobile,” “application,” and “patient” becoming increasingly prominent in recent years. Co-authorship and collaboration networks, however, showed limited cross-national research partnerships.

**Discussion:**

The study highlights the transformative role of digital solutions in pharmacovigilance, demonstrating the potential of ADR applications to enhance reporting accuracy and improve patient safety. However, adoption remains early-stage and fragmented by regional affiliations. Future research should focus on patient-centric app development, effectiveness assessment, and fostering global collaboration. Strengthening digital literacy and robust investment in ADR reporting applications is crucial for optimizing their impact in healthcare.

## Highlights

• Keywords Analysis shows rising interest in “mobile,” “application,” and “patient” in ADR studies.
• NLP-based content analysis identifies ten key themes, including psychiatry and chemotherapy.
• Co-authorship network highlighted limited cross-national research collaborations.
• ADR digital reporting research has grown significantly since 2016, peaking in 2022.

## 1 Introduction

Adverse Drug Reactions (ADRs) are a significant concern in healthcare, leading to mortality, morbidity, and increased costs for healthcare providers. The Global Burden of Disease Study only reported for “adverse effects of medical treatment”, prevalence (2673100) and incidence (34975000) values without describing the main drugs involved.^1^ ADR-related healthcare costs contribute significantly to the overall economic burden, ranging from USD 65.00 to USD 12,129.90 per event.^2^ Therefore, finding effective strategies to enhance the reporting and monitoring of ADRs is crucial in ensuring patient safety, improving healthcare outcomes, and informing regulatory decisions. By actively monitoring ADRs, healthcare professionals can detect patterns, trends, and potential risks associated with specific medications or patient populations. This knowledge can lead to the implementation of appropriate interventions, such as adjusting dosages, issuing warnings, or even withdrawing drugs from the market if necessary.^3^ The existing systems for ADR reporting have limitations, such as underreporting and lack of standardization. For example, a study reported a median underreporting rate was as high as 94 % (interquartile range 82–98 %). Their discovery of significant and pervasive underreporting, especially in cases of severe ADRs, highlights the need for actions to enhance reporting rates.^4^

The rise of mHealth and web-based applications has transformed the health domain, revolutionising the way healthcare is delivered and accessed. These technologies have provided innovative solutions to improve patient care, enhance communication between healthcare providers and patients, and empower individuals to take control of their health.^5^ A comprehensive web and mobile application for monitoring and reporting ADRs can enhance pharmacovigilance practices. It can provide a user-friendly platform for healthcare professionals and patients to report ADRs, ensuring that valuable information is captured and analysed for further investigation.^6^ By harmonizing the reporting process and standardizing the forms used for reporting, the application can improve the rate of reporting and enhance the quality of data collected.^7^ This, in turn, can contribute to the early detection of potential safety concerns, leading to timely interventions and improved patient safety.^8^ Furthermore, a comprehensive web and mobile application can contribute to the overall understanding of ADRs by mapping the existing literature in this field. It can identify research gaps, highlight key themes, and guide future studies and development efforts. The application can also serve as a valuable resource for healthcare professionals, researchers, and policymakers involved in the development and implementation of ADR mobile or web applications.

Despite keen interest from National Regulatory Authorities, healthcare workers and patients in shifting to usage of app-based ADR reporting, there's limited knowledge about the current ADR reporting apps.^9^^,^^10^ Therefore, the primary objective of the study was to provide a comprehensive overview of the studies related to ADR mobile or web applications. To achieve this, a two-stage approach was employed: first, bibliometric analysis was utilized to understand publication trends, key authors, and keyword occurrences in this domain. Second, Natural Language Processing (NLP)-based content analysis was applied to discern the core themes in these studies. Through this combination, a holistic mapping of the existing literature on ADR mobile or web applications is provided. Additionally, the results of content analysis and bibliometric analysis were compared to comprehensively represent the knowledge structure of the domain.

## 2 Methodology

The combination of bibliometric and NLP based content analysis methods provide a robust approach to examining ADR applications. The bibliometric analysis allows for the quantitative/descriptive assessment of research output and performance. On the other hand, the inclusion of NLP-based content analysis is crucial because it enables a more nuanced understanding of the thematic development and the intricate content within the literature. NLP techniques are adept at parsing and analysing large volumes of text, extracting key themes, sentiments, and patterns that might not be evident through traditional analysis methods. This dual approach, merging quantitative bibliometrics with the qualitative depth of NLP, ensures a more thorough and insightful exploration of the field, providing a multi-dimensional understanding that is both broad in scope and rich in detail.^11^ This comprehensive approach contributes to a deeper understanding of the field.

### 2.1 Data collection and pre-processing

Analysis of the scholarly literature in pharmacovigilance domain was conducted to examine the trends and structures in the field. The keywords “pharmacovigilance”, “adverse drug reaction reporting system”, “web or mobile application” were used as search terms. The search was carried out utilizing the Web of Science database, a widely acknowledged and esteemed repository of scholarly literature.

**Fig. 1:**
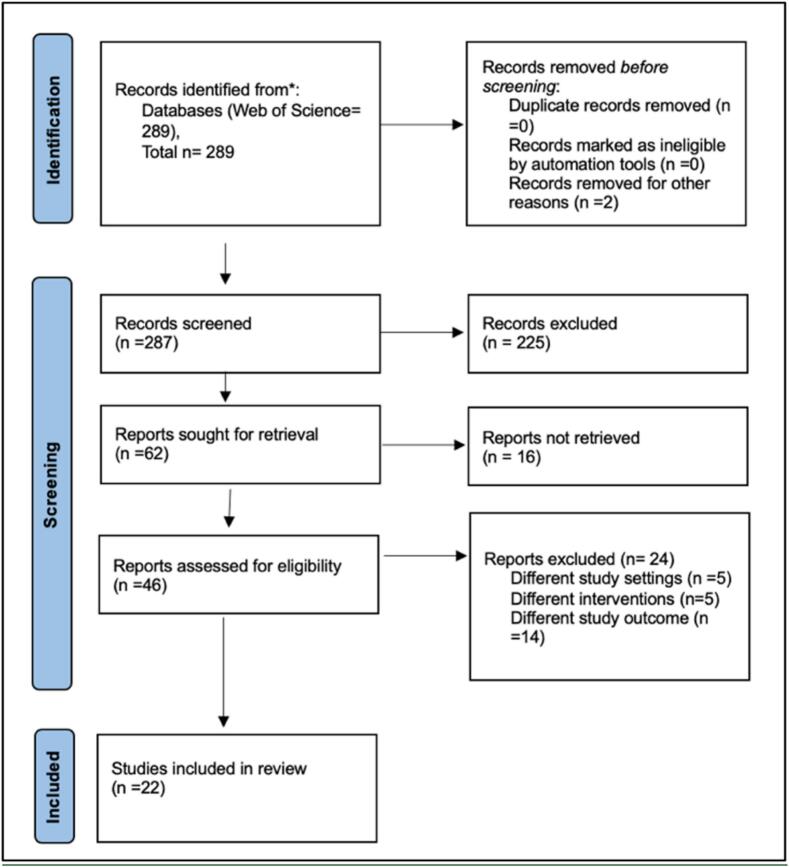
PRISMA flow diagram representing the study selection process.

A total of 289 articles published between 2007 and 2023 were retrieved using this search strategy. Next, the titles and abstracts of documents were examined to determine eligibility based on ‘topical relevance’. Studies that encompassed participants of all age groups, genders, and ethnicities were included. In the context of this research, an “app” is delineated as a software application engineered to operate either on a smartphone or via web platforms. To qualify for inclusion in the study, the application had to include functionalities that allowed users to directly generate an ADR report within the interface, remote surveillance of ADRs, algorithms for detection or prediction of ADRs, and the provision of pertinent information for ADR management. Applications developed in any linguistic medium were considered eligible. This resulted in the exclusion of an additional 184 documents. Of the 47 articles initially considered for full-text screening, 11 articles could not be retrieved. During the full text screening and in conjunction with the previously stated eligibility criteria, studies such as narrative or systematic reviews, editorials, and perspectives were excluded. Therefore, the final database for the content analysis consisted of 22 articles (Fig. 1). Screening for the Title-Abstract (Ti-Ab) stage and the full-text stage was conducted in Rayyan.ai. software.

For computational text analysis, the initial step of analysis involved a comprehensive pre-processing of the collected text data. The raw text was first tokenized, breaking it down into individual words or tokens. This granular representation of the text was essential for the subsequent stages of analysis. Recognizing that certain words, termed as stopwords, such as “and”, “the”, and “is”, often do not contribute significant meaning in the context of topic modelling, these were systematically removed. Additionally, to ensure consistency and avoid redundancy, all words were subjected to stemming or lemmatization. This process reduced words to their root form, treating various forms and derivations of a word as a single entity. For instance, “patients”, “patient”, and “pt” might all be reduced to the root “patient”. The text was also stripped of any special characters and numbers, ensuring that only relevant alphanumeric characters were retained for analysis. Lastly, to maintain uniformity, all characters were converted to lowercase. This rigorous pre-processing phase ensured that the data were primed and ready for effective topic modelling in the subsequent stages of the study.

### 2.2 Data analysis and visualization

#### 2.2.1 Bibliometric analysis

In this paper, the temporal and spatial distribution was analysed through co-authorship analysis and keyword co-occurrence network, overlay visualization and citation analysis. A similar method has been employed in previous literature.^12^^,^^13^ The bibliometric data were processed and analysed using Web of Science, Microsoft Excel and VOSviewer. In the resulting network maps, the nodes represent either authors, publications or keywords. Nodes are linked together to depict relationships or interactions among them. Link strength quantifies the number of connections a node has with other nodes, and the total link strength refers to the sum of these connections. Typically, nodes that are more central or well-connected within the network are positioned closer to the centre of the visualization, while those with fewer connections are situated towards the periphery. Number of documents are used as weights in the visualization or in other words the larger the relative size of a node, larger number of documents are affiliated to the node. A thesaurus was used to eliminate duplicate keywords and the fractional counting was employed. The thresholds for mapping were selected to highlight relevant literature with minimal loss of information. Thresholds were set at least one document and at least one cite for co-authorship analysis and at least two co-occurrences for keyword cooccurrence analysis.

#### 2.2.2 Interactive dashboard

Python's Pandas library was utilized for data pre-processing and NetworkX for network analysis to construct and analyse co-authorship, citation, and country collaboration networks from a scholarly publication dataset containing author names, DOIs, titles, journals, publication years, and country information. Co-authorship networks were constructed, where nodes represented authors and edges denoted co-authorship relations. Citation networks were developed, with documents as nodes and citation links as directed edges. Additionally, country collaboration networks were generated, illustrating international research partnerships. These networks were visualized and interactively explored using Dash Cytoscape, an extension of Plotly's Dash framework, facilitating dynamic user interaction by enabling detailed inspections of node-specific information, such as publication details and collaborative patterns.^14^^,^^15^

#### 2.2.3 Content analysis

##### 2.2.3.1 Computational text analysis

###### 2.2.3.1.1 Feature selection

Once the data were meticulously pre-processed, the next pivotal stage was feature extraction. The objective here was to transform the textual data into a numerical format that could be seamlessly ingested by the topic modelling algorithms. Two primary techniques were used for this transformation: Term Frequency-Inverse Document Frequency (TF-IDF) and Count Vectorization.^16^^,^^17^ TF-IDF is a statistical measure that evaluates the importance of a word in a document relative to a collection of documents (corpus). It assigns higher weights to terms that appear frequently in a given document but are rare across the entire corpus, thereby reducing the influence of common words that may not carry significant meaning. This transformation is particularly useful in emphasizing domain-specific terms while minimizing the impact of frequently occurring generic words.^17^ On the other hand, Count Vectorization converts the textual data into a numerical matrix by counting the frequency of each unique token (word) in the dataset. Unlike TF-IDF, this method does not apply weighting to words based on their global occurrence but instead provides a direct representation of term frequencies in the document corpus. While Count Vectorization retains more raw linguistic information, it does not inherently adjust for the importance of terms across the dataset.^16^

###### 2.2.3.1.2 Model training

With the numerical representation of the text data at hand, the training of topic modelling algorithms was conducted. Two distinct models were employed: Latent Dirichlet Allocation (LDA), and Non-negative Matrix Factorization (NMF).^18^^,^^19^ LDA, introduced by Blei et al. (2003), is a generative probabilistic model that postulates each document as a mixture of a limited set of topics.^18^ Implementing the LDA required specifying the anticipated number of topics present in the corpus. Determining the optimal number of topics can be approached through methods such as coherence score analysis or leveraging domain-specific knowledge. For the estimation of model parameters in LDA, the Variational Bayes method was employed, a technique that approximates the computationally challenging true posterior with a more manageable form.

On the other hand, NMF, a linear algebraic model, factorizes a non-negative data matrix into two lower-rank non-negative matrix factors, highlighting its capacity to extract interpretable parts.^19^ The primary input for NMF was the TF-IDF matrix approach, a representation that captures the significance of terms in individual documents relative to the entire dataset. Similar to LDA, an expected number of topics were defined for the NMF. To bolster the interpretability of the resulting topics, the matrices were normalized, ensuring consistent scaling and potentially accelerating algorithm convergence.

###### 2.2.3.1.3 Model evaluation - topic coherence

Evaluating the effectiveness of the topic models was a crucial step in the methodology. One of the primary metrics used was topic coherence, which gauged the quality of topics produced by LDA model.^20^ A higher coherence score indicated that the words associated with a particular topic were more semantically related and, thus, the topic was more interpretable and relevant.

##### 2.2.3.2 Data Synthesis

As part of the evidence synthesis, data extraction was conducted using a pre-designed Data Extraction Sheet (DES). Relevant data were extracted from the articles for the following: country, population, condition, type of article, web/mobile application, objective and outcome. In case of missing details, no attempts were made to contact authors for the additional details.

## 3 Results

The search on the Web of Science (Clarivate) database yielded 289 studies (Supplementary material 1), of which 62 articles were included for bibliometric analysis, and 22 articles were selected for content analysis. The publication trend from 2007 to 2023 (Supplementary material 1) illustrates a dynamic research landscape. Initially, there was minimal activity in 2007, followed by a noticeable slowdown between 2008 and 2012. However, a substantial increase in publications was observed after 2012, peaking around 2018. A significant decline occurred in 2021, likely influenced by global events such as the COVID-19 pandemic. Yet, 2022 showed a resurgence of interest, indicating evolving research directions and a renewed focus in the domain.

### 3.1 Bibliometric analyses

Based on the search strategy and screening of titles and abstract, 36 relevant publications were identified from Web of Science. Through bibliometric analysis, trends in publication, influential researchers, highly cited work, and key research areas were identified. (https://berbere.pythonanywhere.com/).

#### 3.1.1 Co authorship network

**Fig. 2:**
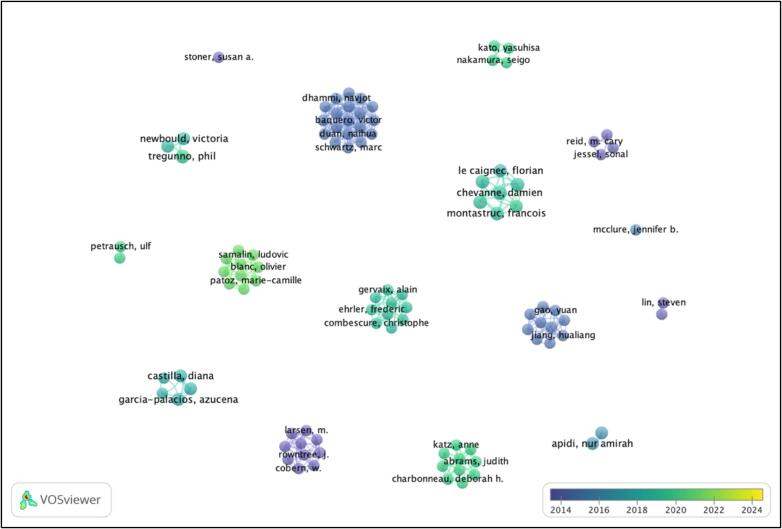
Overlay of networks in studies on ADR mobile and web applications.

Fig. 2 is an overlay image of top 100 authors obtained through fractional counting. The co-authorship analysis shows top 100 authors (Obtained through setting thresholds at, at least one doc and over 12 citations). The nodes in blue are authors with average publication in past years i.e., 2012–2016. The green nodes represent authors who published more articles on an average during 2015–2019 and the yellow nodes represent authors with newer publications who are currently active in the field (2020 onwards). The most productive authors in terms of the number of articles published are Damien Chevanne, Gilles Defer, Sophie Fedrizzi, Francois Montastruc, Jean-Jacques Parienti (Supplementary material 1). They each have three articles with 19 citations. Whereas the authors, with the highest number of citations are Sonal Jessel, Samantha J Parker, M Cary Reid, Joshua E Richardson, each with one document published in the field with 140 citations.

**Fig. 3:**
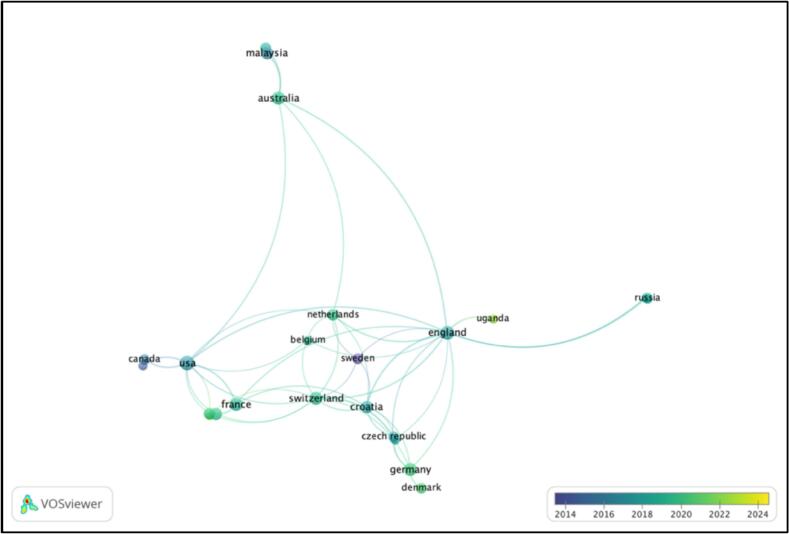
Network visualization mapping for the country of origin of research publications.

Fig. 3 represents a co-authorship network of countries/regions that contributed to ADR research. England and Switzerland are important to the network as nodes with greater centrality. England has the highest total link strength of nine and has published the most articles (*n* = 10) whereas Switzerland has total link strength of five. Of 29 countries that published articles in the field, only 20 countries have published at least two articles. The highest cited articles are from US (336), England (174) and Switzerland (106).

#### 3.1.2 Keywords Co-occurrences

**Fig. 4:**
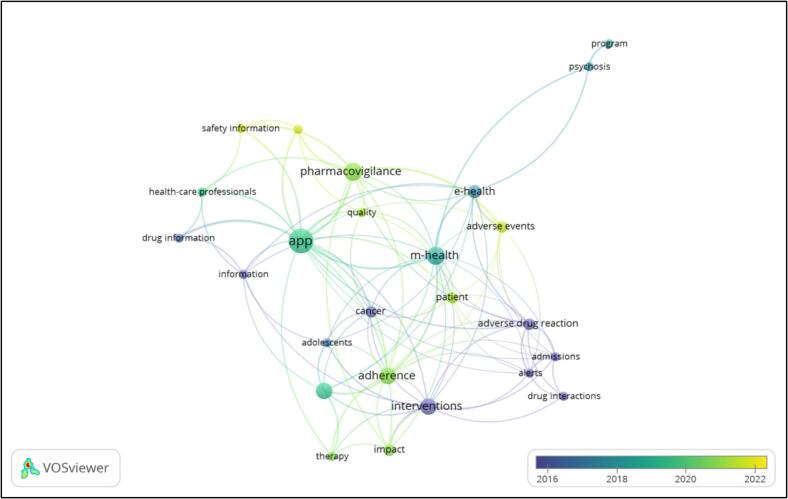
Network visualization mapping for all keywords.

Fig. 4 is the network visualization mapping for the keywords. The trends in the field are indicated over the years by colour gradient. Of 321 keywords in the network, 64 are co-occurring more than once. Key words with highest total link strength are apps, m health, pharmacovigilance, interventions, adherence. Smartphone, e-health, adverse drug reaction, adverse events, cancer, impact, and patient are occurring at least three times.

### 3.2 Content analysis

Following the bibliometric analysis, NLP-based content analysis was employed to delve deeper into the data. This approach enabled a systematic analysis of the textual content of identified publications, allowing for the extraction of thematic patterns, and key terminologies prevalent in the field. A total of 22 articles were included for the content analysis.

#### 3.2.1 Yearly topic distribution

In the presented word cloud visualization (Supplementary material 1), the prominence of specific terms across distinct years is depicted. The size of each term is proportionate to its frequency, offering insights into the dominant themes or subjects that surfaced annually. Notably, terms such as mobile, application, adverse and patient have gained significant prominence in recent years, emphasizing growing interest in these areas.

#### 3.2.2 Themes derived from the literature using topic modelling

To achieve a comprehensive understanding of the existing literature, ten key themes were identified based on using three distinct topic modelling techniques (Table 1). Obtained Coherence Score of 0.75 signifies a commendable level of topic coherence, suggesting that the topics delineated by the model are semantically consistent and contextually pertinent. This score emphasizes that the terms within each topic exhibit a frequent co-occurrence pattern across the dataset.

**Table 1:** Themes and keywords derived from topic modelling.

Theme	LDA keywords	NMF Keywords
Pharmacovigilance, safety and reporting	app, reporting, mobile, adr, web, patients, radr, safety, apps, patient	mobile, adverse, patient, reports, pharmacovigilance, patients, drug, adrs, reporting
Chemotherapy and Treatment Adherence	adherence, participants, study, program, cancer, medication, oral, intervention, patient	chemotherapy, intervention, participants, sms, program, oral, cancer, adherence, oral chemotherapy
Specialty area: Psychiatry Research	app, patients, health, patient, mobile, depression, treatment	users, webradr, psychiatry, side effects, study, reporting, depression, reports, apps
Patient Health and digitisation	patients, cancer, health, mhealth, pain, apps, patient, study, application, therapy	headache, mobile, healthcare, clients, clinicians, mhealth, application, server
Mobile Alerts for Severe Medical Symptoms⁎	–	mobile, syndrome, phone, temperature, nurse, moderate, vomiting, red, severe, alerts
Drug Interactions in the Elderly⁎	–	advice, elderly, drugs, risk, web, interactions, criteria, interaction, drugdrug, drug
Specialty area: TB Reporting⁎	–	mobile, app-based, participants, prototype, tb, study, reporting, reports, app
Specialty area: Dermatology⁎	–	image, dermatology, uncertain, postlaser, photos, smartphone
Clinical and research applications⁎	clinical, patients, study, treatment, cal, related, medication, health, bd	–
Comprehensive and accessible health Information resources⁎	drug, online, resources, mobile, health, apps, com, drugs, cam, interactions	–

⁎ In this table, blank columns result from the differing mechanisms of LDA and NMF. LDA captures broader themes, whereas NMF is more specific, leading to non-uniform keyword extraction.

In the domain of ‘Pharmacovigilance, safety and reporting’, there is a discernible emphasis on modern tools such as app, reporting, and mobile, highlighting the increasing integration of technology in pharmacovigilance practices. The theme ‘Chemotherapy and Treatment Adherence’ underscores the crucial aspects of adherence, chemotherapy, participants, and study, shedding light on the factors influencing patient adherence during treatment. The specialty area of ‘Psychiatry Research’ underscores the utilization of app, patients, and health as pivotal elements, indicating a trend towards digital tools in mental health research. In the realm of ‘Patient Health and digitisation’, there is a pronounced focus on patients, clinicians and health, pointing to the digital transformation of patient health management. Other themes, such as ‘Specialty area: TB Reporting’ theme brings attention to the significance of mobile, app-based reporting, and participant feedback in the context of Tuberculosis control and ‘Specialty area: Dermatology’, there is a focus on image analysis, dermatological conditions, and diagnostic uncertainties. Lastly, the theme ‘Comprehensive and accessible health Information resources’ accentuates the need for drug databases, online platforms, and resource accessibility in today's healthcare landscape.

#### 3.2.3 Characteristics of included studies

Table 2 presents extracted data from the included studies. Several applications have been developed with distinct functionalities tailored to different clinical needs. A predominant category of these applications allows patients to directly report specific ADRs, enhancing real-time monitoring and response. For instance, some applications are specifically designed for patient groups, such as breast cancer patients under hormone therapy, ensuring that the unique ADRs experienced by these groups are adequately captured and addressed. Remote patient monitoring is another innovative approach facilitated by these applications. Patients can download specific apps, take standardized images of their treatment areas, and subsequently enable clinicians to remotely monitor potential side effects and skin reactions. This not only ensures continuous patient monitoring but also minimizes frequent hospital visits. The “Med Safety” application exemplifies the proactive approach in ADR reporting. Its primary objective is to augment the ADR reporting rate among healthcare professionals attending to patients with specific treatments. The post-vaccination period is crucial, and recognizing this, dedicated applications have been established to document adverse events following immunizations. The utility of these applications is not limited to just reporting; features enabling symptom and side effect tracking over time are integral in some of these tools. Certain prototypes have been developed, underscoring the potential for enhanced ADR reporting or comprehensive understanding of medication side effects. A salient feature of systems is the alert mechanism; upon detecting ADRs, these systems promptly notify professionals, facilitating timely interventions.

**Table 2:** Characteristics of included studies.

Country	Type of article	Condition	Population	Web/mobile application	Objective/Use of application	Outcome
Turkiye^21^	Cross-sectional	General	Geriatric population	Web application	Developed a database for geriatric patients to assess drug appropriateness	Identified high rates of polypharmacy, multimorbidity, and PIM, with time-saving benefits for analysis
France^22^	Study protocol	Breast cancer	Patients	Web application	Enabled ADR reporting for breast cancer patients on hormone therapy	Improved ADR management and personalized monitoring
Denmark^23^	Proof-of-concept study	Psoriatic disease	Patients	Mobile application	Smartphone app for remote monitoring of psoriatic disease treatment	Provided evidence on Cal/BD foam treatment, OCT-guided AFL pre-treatment, and reduced clinic visits via smartphone-based monitoring
Uganda^24^	Study protocol	HIV with TB	Patients	Mobile application	Med Safety app for ADR reporting by healthcare professionals	Increased ADR reporting through training in mobile, paper-based, and web-based methods
Australia^25^	Proof of concept trial	Cancer	Patients	Mobile application	Evaluated intervention on adherence to oral chemotherapy (OC)and side effects	The smartphone program designed to support adherence to OC showed high acceptability and satisfaction among OC users
Brazil^26^	Pilot study	Malaria	Patients	Mobile application	ADR reporting for the Brazilian Health Regulatory Agency	Haemolytic anaemia symptoms were reported in 5.37 % of patients; three G6PD deficiency cases identified
Germany^27^	Cross-sectional	COVID-19 vaccination	Community	Mobile application	Mobile app for documenting adverse events post-vaccination	Strengthened patient empowerment and self-monitoring in healthcare
France^28^	RCT	Multiple sclerosis	Patients	Mobile application	Mobile app for ADR reporting in multiple sclerosis patients	Increased ADR reporting for disease-modifying treatments
US^29^	Cross-sectional	Cancer	Patients	Mobile application	Symptom and side-effect tracking for cancer patients	Highlighted gaps in patient engagement and self-monitoring in cancer apps
India^30^	Descriptive study	General	Community	Mobile application	Mobile app to track TB treatment progress and symptoms	Aimed to optimize doctor follow-ups and address treatment administration issues
Germany^31^	Cross-sectional	Influenza vaccination	Community	Mobile application	Prototype app for influenza vaccine AEFI reporting	Demonstrated feasibility of app-based AEFI reporting and feature identification
Brazil^32^	Pilot study	General	Community	Mobile application	Mobile app for monitoring medication importance and side effects	Enhanced patient participation in managing peripheral arterial disease
Australia^33^	Descriptive study	Cancer	Patients	Mobile application	App for managing chemotherapy side effects	Achieved over 80 % adherence rates and high user satisfaction
The Netherlands^34^	Cross-sectional	General	Community	Mobile application	Mobile app for spontaneous ADR reporting	Attracted a higher proportion of patient-reported ADRs than conventional methods
France^35^	Study protocol	Relapsing-remitting multiple sclerosis	Patients	Mobile application	Mobile app for ADR reporting in multiple sclerosis	Expected to increase patient ADR reporting tenfold
Iraq^36^	Pilot study	Headache	Patients	Mobile application	Mobile app for recording headache-related side effects	Improved patient safety, real-time monitoring, and healthcare integration
Australia^37^	Retrospective study	Facial laser resurfacing	Patients	Mobile application	Smartphone app for self-reporting side effects post-laser resurfacing	Identified barriers and benefits of smartphone-based post-treatment monitoring
Croatia^38^	Descriptive study	Patients taking antipsychotic medication	Patients	Mobile application	App for early detection of antipsychotic medication side effects	Provided detailed tracking with severity ratings for 30+ side effects
US^39^	Study protocol	Chronic pain	Patients	Mobile application	System for reporting unanticipated ADRs to the Safety Monitoring Committee	Evaluated impact on pain-related daily functioning over 26 weeks
US^40^	Descriptive study	General	Community	Web application	VAMedSAFE web application for monitoring ADR risks	Improved prescribing and monitoring of erythropoiesis-stimulating agents (ESAs)
Sweden^41^	RCT	General	Community	Web application	Software for categorizing DDIs by clinical significance	Found 16 % of significant DDIs to be clinically relevant, highlighting prioritization needs
UK^42^	Feasibility study	Colon cancer	Patients	Mobile application	Mobile system for self-reporting ADRs in colon cancer patients	Strengthened patient engagement and confidence in symptom tracking, with minimal reported side effects

AEFI: Adverse Events Following Immunization, DTG-based cART: Dolutegravir-based combination antiretroviral therapy, PIM: Potentially Inappropriate Medicines, VAS: Visual Analogue Scale, DDI: Drug-Drug Interaction.

## 4 Discussion

Through comprehensive bibliometric analysis of 36 pertinent publications, salient trends in publication, key researchers, and principal research domains were discerned. Notably, a co-authorship network, demarcated the authors in this field using fractional counting, categorizing them based on their publication periods: authors with predominant publications during 2014–2016, those more active between 2016 and 2018, and emerging contributors currently active in the field. Highlighted contributors include Chevanne, Damien, and Defer, Gilles, among others. The content analysis, visualized through a word cloud, underscores the escalating prominence of terms like “mobile,” “application,” “adverse,” and “patient” over recent years. A deep dive into the extant literature using topic modelling techniques identified ten pivotal themes including pharmacovigilance, safety and reporting, chemotherapy and treatment adherence, specialty area: psychiatry research.

The results portray the increasing use of digital solutions to address ADR challenges, particularly with regards to underreporting.^43^ While the study did not confirm whether the enhanced use of mobile or web applications has led to a significant global shift in ADR reporting, the content analysis identifies the increasing linkage between pharmacovigilance, patient safety and digital applications. Additionally, the co-authorship analysis highlighted that, much of the literature on apps for ADR is associated with nations with a robust pharmaceutical industry presence, such as the UK, Switzerland, US, Australia, and various European countries.^44^ The study networks are indicative of the precursor stage of research as no author or team of researchers has yet had a decisive influence on the field, which is also related to the fact that the field is still in the precursor stage of development. Furthermore, there seems to be a strong tendency for members within these networks to align more closely based on institutional or national affiliations, exhibiting limited cross-national or interregional collaboration.

Several themes emerged through topic modelling analysis. Notably, the prominent themes highlight, ‘Drug Interactions in the Elderly,’ as well as specific focus areas such as ‘Dermatology’ and ‘TB Reporting,’ as well as the critical issue of ‘Cancer, Chemotherapy and Treatment Adherence.’ Each of these themes accentuates the multifaceted nature of ADRs across various patient demographics and specialty areas, shedding light on the nuanced challenges that healthcare providers confront when addressing adverse reactions.

Studies in the past have consistently highlighted the elderly's susceptibility to drug interactions, attributed largely to polypharmacy and age-related physiological alterations.^45^^,^^46^ The dermatological dimension brings to the fore the myriad drug interactions manifesting and being reported as cutaneous reactions, a facet corroborated by previous studies.^47^ Meanwhile, the emphasis on chemotherapy adherences reflects current literature, which frequently emphasizes the dire consequences of non-adherence, especially in the face of potential drug interactions.^48^ The TB reporting facet resonates strongly with global health priorities, particularly with the ongoing battle against multi-drug resistant TB strains.^49^ The emergence of these themes suggests not only the expanding scope of ADR-focused digital solutions but also accentuates the urgent imperative to address these specific areas to improve patient outcomes. The study's findings highlight the significant role of web and mobile applications in modern medical practices. These digital tools have demonstrated their effectiveness in enhancing patient-reported outcomes, promoting treatment adherence, and efficiently monitoring side effects. This suggests a considerable positive impact on patient safety and healthcare quality. The growing reliance on and importance of these digital solutions in healthcare settings is evident, marking a pivotal shift towards more patient-centered and technologically integrated medical care.^50^^,^^51^

The study revealed that the evidence is still in its nascent stages with wide scope of enquiry for future research. Consequently, specific areas of exploration are advocated for in forthcoming research. Evidence on the impact of the digital technology in reducing ADR needs to be generated on priority from medical researchers. Leveraging the widespread digital literacy and pervasive use of smart devices could herald a transformative era.^52^ Not only can this combination aid in the prompt identification of ADRs, but it can also serve as a pivotal tool in in assessing the appropriateness of medications, considering various risk factors such as gender, race, and the presence of co-morbidities.^53^ Further, it is paramount that investments in these apps or solutions are underpinned by credible information on its usage, uptake and impact. Determining which app specifications cater optimally to which user subgroups should be prioritised. Although the march towards digitization seems inevitable, it's pivotal to carve out an effective strategy for data collation and subsequent action plans, ensuring that we harness the true potential of this digital transition.

Like other studies, the present study is not without its limitations. A notable limitation is the predominant reliance on English language sources and a singular database. This potentially excludes publications in other languages (such as Chinese and French) and those available in alternative databases. Future research could enhance the comprehensiveness by incorporating documents in various languages and by accessing multiple databases. Moreover, this study employed a select array of methods intrinsic to science mapping. Fellow researchers are encouraged to explore this methodology, either for a holistic overview of the knowledge domain or for specific thematic assessments. Despite its limitations, the current study demonstrates considerable strengths through its methodological approach. The employment of science mapping methods and content-based analysis in this study offers a systematic and comprehensive overview of the knowledge domain. These methods facilitate a detailed understanding of both the broad trends and specific thematic areas within the field.

## 5 Conclusion

The study utilises unique methodology to describe the field of ADR apps in scientific literature. The analysis indicates that while publication output is trending upward, significant knowledge gaps remain, reflecting the precursor stage of development. This research is particularly timely, as the digitisation of health information is being rapidly and rampantly utilized to enhance ADR management and prevention.

## Ethics approval and consent to participate

Not required.

## Funding

This study did not receive any specific grants from funding agencies in the public, commercial, or non-profit sectors.

## CRediT authorship contribution statement

**Viola Savy Dsouza:** Writing – original draft, Visualization, Methodology, Formal analysis, Data curation, Conceptualization. **Lada Leyens:** Writing – review & editing, Supervision, Investigation, Conceptualization. **Angela Brand:** Writing – review & editing, Supervision.

## Declaration of competing interest

The authors declare that they have no known competing financial interests or personal relationships that could have appeared to influence the work reported in this paper.

## Data Availability

All data that were synthesized or analysed during this study are included in this article.

## Appendix A Supplementary data

Supplementary material 1 Image 1
