# Supplementary material for: Employing bibliometrics and natural language processing (NLP) to analyse real-world applications of adverse drug reaction
Source: Explor Res Clin Soc Pharm. 2025 Mar 17;18:100592. doi: 10.1016/j.rcsop.2025.100592 (PMC11979945; doi:10.1016/j.rcsop.2025.100592)

**[Supplementary Material 1](https://www.sciencedirect.com/science/article/pii/S2667276625000198#ec0005)**

Figure 1: Publication trend


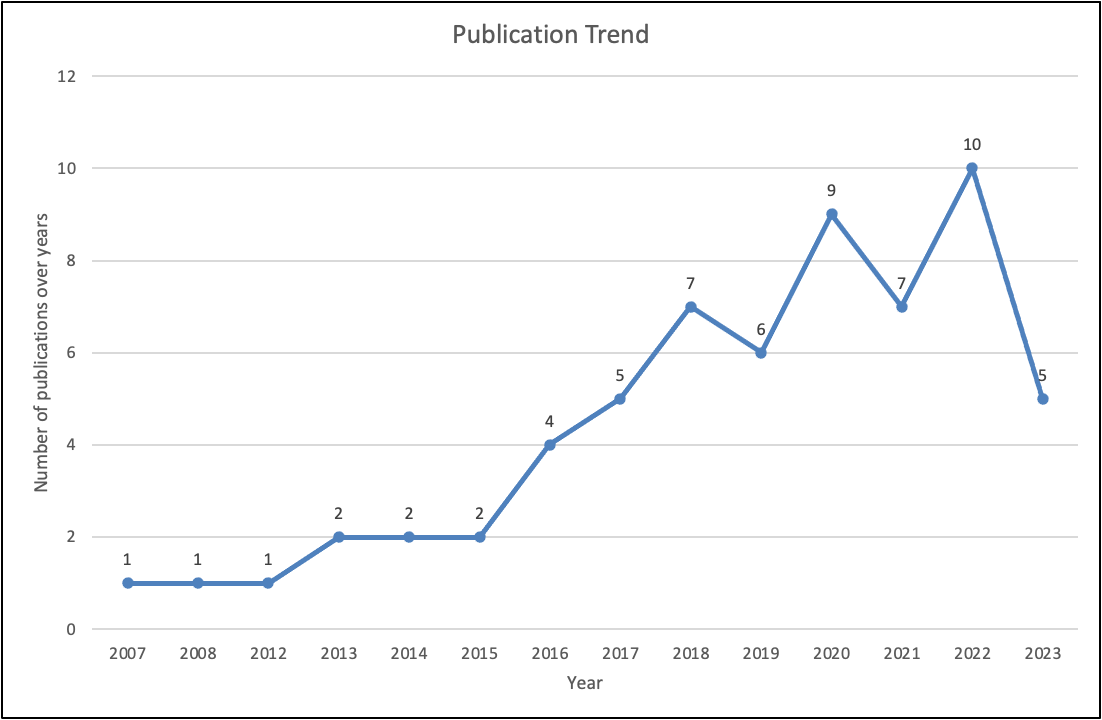


Figure 2: The largest connected network of authors shows specific authors who are linking different clusters


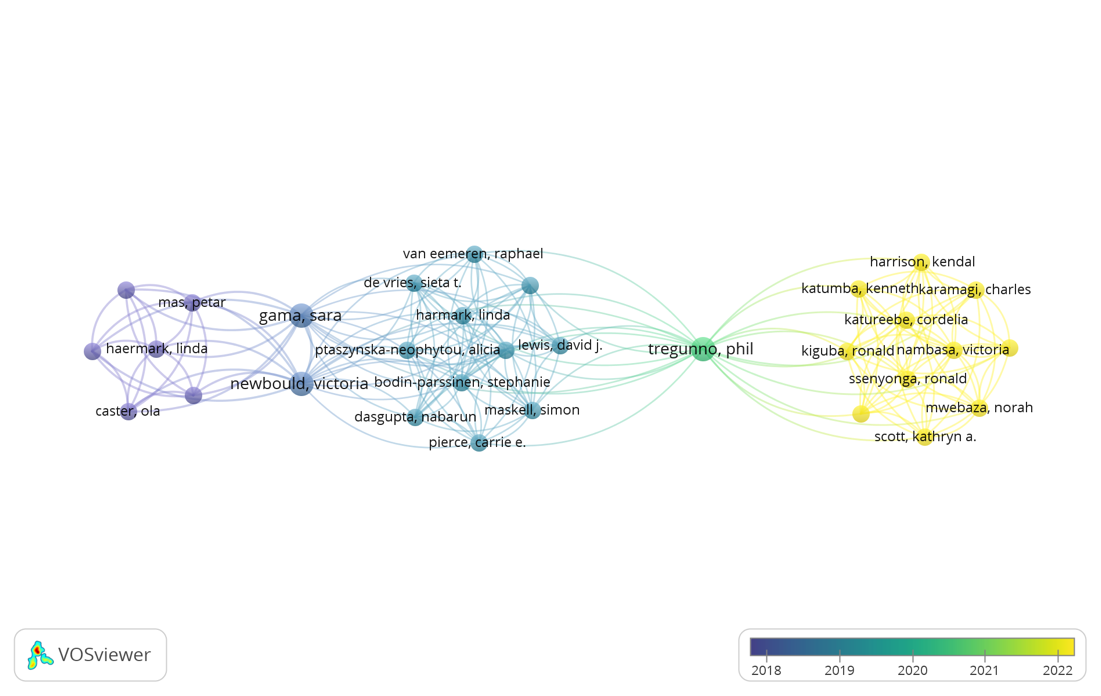


.

Figure 3: Word clouds representing the Yearly Topic Distribution for the years 2016 to 2020


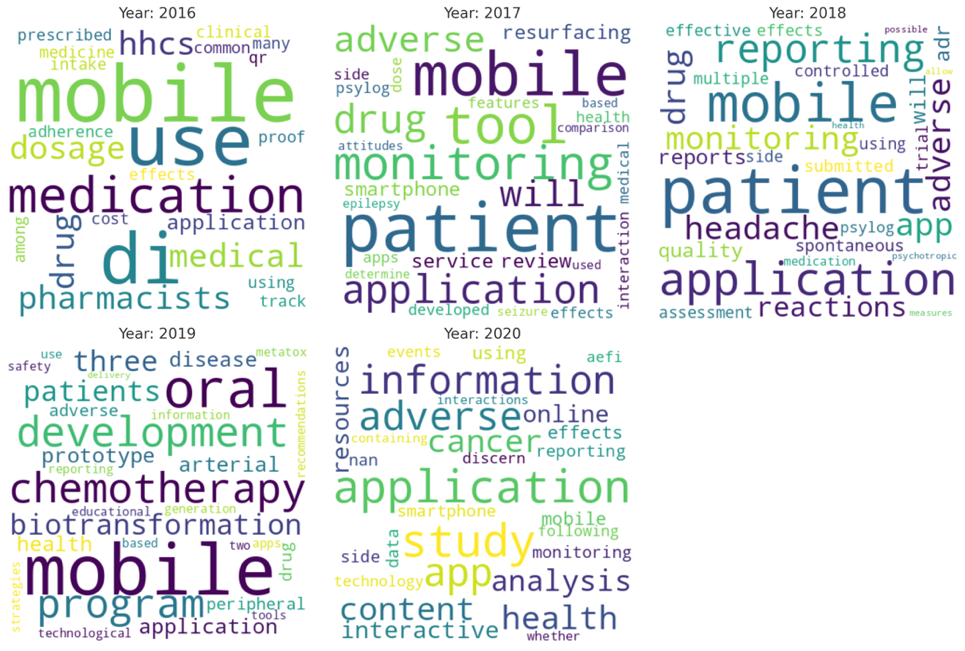

Supplement: Supplementary file 1 — Supplementary material 1 [file mmc1.docx]
